# Stimuli-specific senescence of primary human lung fibroblasts modulates alveolar stem cell function

**DOI:** 10.21203/rs.3.rs-3879423/v1

**Published:** 2024-01-29

**Authors:** Nora Bramey, Maria Camila Melo-Narvaez, Fenja See, Beatriz Ballester-Lllobell, Carina Steinchen, Eshita Jain, Kathrin Hafner, Ali Önder Yildirim, Melanie Königshoff, Mareike Lehmann

## Abstract

Aging is the main risk factor for chronic lung diseases (CLDs) including idiopathic pulmonary fibrosis (IPF) and chronic obstructive pulmonary disease (COPD). Accordingly, hallmarks of aging such as cellular senescence are present in different lung cell types such as fibroblasts in these patients. However, whether the senescent phenotype of fibroblasts derived from IPF or COPD patients differs is still unknown. Therefore, we characterized senescence at baseline and after exposure to disease-relevant insults (H _2_ O _2_ , bleomycin, and TGF-β1) in cultured primary human lung fibroblasts (phLF) from control donors, IPF, or COPD patients. We found that phLF from different disease-origins have a low baseline senescence. H _2_ O _2_ and bleomycin treatment induced a senescent phenotype in phLF, whereas TGF-β1 had primarily a pro-fibrotic effect. Notably, we did not observe any differences in susceptibility to senescence induction in phLF based on disease origin, while exposure to different stimuli resulted in distinct senescence programs in phLF. Moreover, senescent phLF reduced colony formation efficiency of distal alveolar epithelial progenitor cells in a stimuli-dependent manner. In conclusion, the senescent phenotype of phLF is mainly determined by the senescence inducer and impairs alveolar epithelial progenitor capacity *in vitro* .

## Introduction

Chronic respiratory diseases are the third leading cause of death globally [1]. Those include chronic obstructive pulmonary disease (COPD) and interstitial lung diseases such as idiopathic pulmonary fibrosis (IPF) [1]. COPD is an inflammatory disease [2] characterized by small airway remodeling, emphysema, and chronic bronchitis [3]. The main risk factors are cigarette smoking and age but exposure to air pollution or pathogens also contribute to COPD [3]. IPF is a progressive fibrosing disease characterized by excessive matrix deposition [4, 5]. Higher age and exposure to cigarette smoke are the main risk factors for IPF [4, 5]. Familial cases of IPF have mostly been linked to mutations in genes encoding surfactant protein C and A2 (SFTPC, SFTPA2) and telomerases (TERT and TERC), which lead to telomere shortening, cellular senescence, and exhaustion of lung stem cells [4–7]. The incidence rates for both COPD and IPF increase in the elderly population [8] and several cellular hallmarks of aging such as cellular senescence are increased in the lung tissue from both COPD and IPF patients [9–12]. Senescent cells are characterized by an irreversible cell cycle arrest, resistance to apoptosis, altered metabolism and the secretion of growth factors and pro-inflammatory cytokines known as the senescence associated secretory phenotype (SASP) [13].

Fibroblasts are effector cells in both diseases, causing impaired tissue structure by aberrant deposition of extracellular matrix (ECM) on the one hand, and demonstrating hallmarks of senescence on the other hand [10, 11, 14–16]. Recent progress in single cell omics revealed the existence of disease-specific cellular subtypes in the mesenchymal compartment [17–21]. Although accumulation of senescent cells has been shown in both COPD and IPF, it remains unclear how senescence is induced in specific cell types and whether certain subtypes are more prone to different senescence stimuli. Therefore, here we used well-known senescence inducers to study susceptibility and senescence programs in primary human lung fibroblasts (phLF) from control donors, IPF, and COPD patients. In this study, we show that phLF from different disease origins have a low baseline senescence in culture. Moreover, stimuli and not disease origin determines the senescence phenotype in phLF. Finally, senescent fibroblasts modulate progenitor cell capacity of alveolar epithelial progenitors *in vitro*.

### Materials and methods

## Ethic statement

The study was approved by the local ethics committee of the Ludwig-Maximilians University of Munich, Germany (Ethic vote 19–630). Written informed consent was obtained for all study participants.

## Cell culture

Primary human lung fibroblasts (phLF) from age-matched control, COPD, and IPF patients (Table 1) were obtained from the CPC-M bioArchive at the Comprehensive Pneumology Center (CPC Munich, Germany). PhLF were isolated [22] and cultivated in DMEM/F-12 (Life Technologies, USA) with 1% penicillin/streptomycin (10.000U/ml, Life Technologies, USA), and 10% Fetal Bovine Serum (PAN Biotech, Germany) and medium was changed every second day. For qPCR, Western Blot, and ELISA experiments, phLF were seeded on 6-well plates at a density of 4.5 x $10^4$ cells per well. After 24h of incubation, treatment solutions were applied and changed every 48h. After day3 and day7 of treatment, supernatants of cells were collected, centrifuged, and frozen at -80°C. For RNA isolation, treatment solution was removed, the wells were washed twice with DPBS and cells were frozen directly at -80°C.

## Induction of cellular senescence

PhLF (passages 3–9) were exposed to 5ng/ml recombinant human TGF-β1 (R&D Systems, 240-B-002), 180μM $H_2O_2$ (Sigma, Germany) or 3.3mU/ml bleomycin sulfate (Sigma, Germany) in culture medium with 5% FBS and treatment was replenished every 48h. Negative control solutions contained equivalent volume of 0.1% BSA in PBS (TGF-β1), plain medium ($H_2O_2$), or DPBS (bleomycin).

Table 1
Patient demographics and clinical data

| Gender | Age (Mean + SD) | Age | Smoker | Diagnosis |
|---|---|---|---|---|
| female | 70+/-11.2 | 72 | ex-smoker > 6 months | Lung cancer/Peritumor |
| female | | 67 | smoker | Lung cancer/Peritumor |
| male | | 84 | ex-smoker > 6 months | Lung cancer/Peritumor |
| female | | 57 | NA | |
| female | 63.6+/-8.8 | 56 | ex-smoker | IPF, no PAH |
| male | | 72 | unknown | IPF, no PAH |
| female | | 54 | never smoker | ILD collagenosis, no PAH |
| male | | 73 | unknown | IPF, no PAH |
| male | | 63 | ex-smoker > 6 months | IPF, precapillary PH |
| male | 63.7+/-10.1 | 67 | ex-smoker > 6 months | COPDIV |
| male | | 73 | ex-smoker > 6 months | COPDIV; no PAH |
| female | | 69 | ex-smoker | COPDIV; low PAH |
| male | | 62 | ex-smoker | COPDIV; Emphysema |
| female | | 47 | unkonwn | COPDIV |

## Organoid assay

Murine distal lung epithelial cells (CD45-/CD31-/EpCAM+) were obtained after enzymatic and mechanic digestion of mouse lungs [23] and MACS sorting. phLF were treated as previously described and after 7 days, control and senescent phLF were treated with Mitomycin C (10 µg/ml) for 2h, at 37°C and 5% $CO_2$ to stop proliferation. Then, phLF were washed with 1X DPBS and kept in fresh medium for at least 1h at 37°C and 5%$CO_2$. phLF and murine distal lung epithelial cells were mixed in a 1:1 ratio (10.000 cells each) in Matrigel (Corning, USA) and seeded on 96-well plates as previously described [23]. Organoid medium supplemented with Rock inhibitor (Ri) was added only for the first 48 h and after this, medium was changed every 2–3 days [23]. After 14 days, organoids were imaged using a LifeCellImager Observer Z1 (Zeiss, Germany). Maximum projections were generated on Zen software (Zeiss, Germany). Organoid size and number were determined using the Napari organoid counter [24, 25]. Data and plots were generated and analyzed in GraphPad Prism 9.5.1.

## RT-qPCR

RNA was isolated using the peqGOLD Total RNA Kit (VWR) according to manufacturer's instructions. For cDNA synthesis, mastermix was prepared by mixing: Random Hexamers (10uM, Invitrogen, cat.#

100026484), dNTP Mix (2mM, Thermo Scientific, cat.# R0192), 5x First-strand buffer (1X, Invitrogen, cat.# Y02321), 0.1 M DTT (40mM, Invitrogen, cat.# Y00147), RNAse Inhibitor (4U/ul, Applied Biosystems, cat.# N8080119), and M-MLV Reverse Transcriptase (10U/ul, Invitrogen, cat.# 28025013) using the following reverse transcription program: 1 cycle at 20°C for 10min, 1 cycle at 43°C for 75min, and 1 cycle at 99°C for 5min. RT-qPCR mix was prepared using 1X Light Cycler 480 SYBR Green Master (Roche, Germany) and primer pairs at 5uM (Table 2). Then, samples were run in a Light Cycler 480II (Roche, Germany): 1 cycle at 50°C for 2 min, 1 cycle 95°C for 5 min, followed by 45 cycles of 1X 95°C for 5s, 1X 59°C for 5s, 1X72°C for 5s. The dCt values were determined by a two-derivative method and log2FC were calculated based on the $2^{-\Delta\Delta C}$ method [26].

Table 2
Primers used for RT-qPCR.

| Primer | Sequence (5´-3´) |
|---|---|
| ACTA2_fw | CGAGATCTCACTGACTACCTCATGA |
| ACTA2_rv | AGAGCTACATAACACAGTTTCTCCTTGA |
| FN-1_fw | CCGACCAGAAGTTTGGGTTCT |
| FN-1_rv | CAATGCGGTACATGACCCCT |
| COL1a1_fw | CAAGAGGAAGGCCAAGTCGAG |
| COL1a1_rv | TTGTCGCAGACGCAGATCC |
| PAI-1 fw | GACATCCTGGAACTGCCCTA |
| PAI-1 rv | GGTCATGTTGCCTTTCCAGT |
| CDKN2A_fw | ACCAGAGGCAGTAACCATGC |
| CDKN2A_rev | CCTGTAGGACCTTCGGTGAC |
| CDKN1A_fw | GTCAGTTCCTTGTGGAGCCG |
| CDKN1A_rev | TGGGTTCTGACGGACATCCC |
| TP53_fw | CGCTTCGAGATGTTCCGAGA |
| TP53_rv | CTTCAGGTGGCTGGAGTGAG |
| HPRT_fw | AAGGACCCCACGAAGTGTTG |
| HPRT_rv | GGCTTTGTATTTTGCTTTTCCA |

## ELISA

ELISA for IL-6 (DY206), GDF-15 (DY957), Serpin E1/PAI-1 (DY1786), and total MMP-3 (DY513) were done according to manufacturer’s instructions (R&D Systems, USA). Absorbance was measure at 450nm using the microplate reader Sunrise (Tecan GmbH, Germany). Final absorbance values were calculated by subtracting the background signal. Concentrations were calculated by interpolation of a linear regression based on the standard curve. Concentrations were normalized to total protein content of the cell lysate at the final collection time point. Heatmaps display the mean of fold changes (treatment/control) of at least 3 biological replicates.

## Senescence associated β-Galactosidase staining phLF were seeded ($8.0x10^3$ cells/well) and the Senescence β-galactosidase staining Kit from Cell Signaling (9860) was used according to manufacturer's instructions to determine senescence induction after day3 and day7 of treatment. **Immunofluorescence staining**

Organoids were fixed with ice-cold methanol and 2D cell cultured cells were fixed with 4%PFA. Then, samples were blocked with 5% donkey normal serum in 0.1% PBST for 1h at RT, and then incubated with primary antibodies (Table 3) diluted in 1% donkey normal serum at 4°C overnight. Samples were washed 3X for 20 min (organoids) or 5 min (cells) with 0.1% PBST and then incubated for 2h at RT with secondary antibodies (Table 3) plus DAPI. Cells were then washed again 3 times and mounted with fluorescence mounting medium (Dako). Mean fluorescence intensity of images taken with a LSM 710 Confocal microscope (Zeiss, Germany) were quantified using ImageJ (Fiji).

## Data collection and analysis

Primary human fibroblasts from Donor, COPD, and IPF were used for different experiments in this study (Table 1). For titration (Fig. 2) and organoid assays (Fig. 6) phLF only from control donors were used and single points represent different biological or technical replicates as indicated in figure legends. To analyze the capacity of the triggers used to induce senescence (Fig. 3) we pooled together the data collected using all the different samples listed in Table 1. Here, single points represent biological replicates and points shape indicate the disease origin. Finally, to study differences linked to the background disease we separated the samples listed in Table 1 into three different groups and used the data collected with these same samples for downstream analysis. Here, single points represent biological replicates and points shape indicate the disease origin.

Table 3
Antibodies used for immunofluorescence stainings.

| Target protein | Host | Company | Ref. No |
|---|---|---|---|
| P21 | rabbit | Abcam | ab109520 |
| Phospho-histone H2A.X | mouse | Millipore | 05-636 |
| ACT | mouse | Abcam | ab24610 |
| SP-C | rabbit | Abcam | ab3786 |
| Krt8 | rat | DSHB | TROMAI |
| Anti-rat-488 | donkey | Invitrogen | A21208 |
| Anti-rabbit-647 | donkey | Invitrogen | A31573 |
| Anti-mouse-568 | donkey | Invitrogen | A10037 |
| Anti-mouse-555 | goat | Invitrogen | A21424 |
| Anti-rabbit-488 | goat | Invitrogen | A11008 |

### Results

## Primary human lung fibroblast isolated from different lung diseases exhibit similar senescence phenotypes at baseline

The phLF derived from Donor, COPD, or IPF patients were characterized at baseline for multiple well-accepted senescence markers after short (Day3) and prolonged (Day7) culture (Fig. 1A). First, we evaluated the senescence-associated-β (SA-β)-galactosidase activity and observed a low percentage of SA-β-galactosidase + cells in all three groups with no significant differences among them (Fig. 1B). Similarly, we did not observe any significant difference in the proliferation rate as determined by cell counts ratio (Day7/Day3) (Suppl. Figure 1A). The expression of the cyclin dependent kinase inhibitor 1A (*CDKN1A/P21*) and 2A (*CDKN2A/P16*) and the tumor suppressor protein 53 (*TP53*) was determined. For all conditions, we observed an upregulation of *CDKN1A/P21* and *CDKN2A/P16* with no statistical difference among the different origins (Fig. 1C). Finally, we found no significant differences in the secretion of the SASP components: Plasminogen Activator Inhibitor 1 (PAI-1), Matrix Metalloproteinase-3 (MMP-3), Growth Differentiation Factor 15 (GDF-15), or Interleukin 6 (IL-6) by phLF from Donor, COPD, or IPF tissue, respectively (Fig. 1D) but we did observe an increase in GDF-15 and IL-6 secretion for all origins from day3 to day 7. In conclusion, we observed a low level of senescence-related markers in cultured phLF irrespective of their disease/non-disease background.

## Induction of senescence in primary human fibroblasts with different stimuli

Aging and exposure to cigarette smoke are the main risk factors for IPF and COPD and have been linked to stress-induced senescence. Therefore, we exposed phLF to hydrogen peroxide ($H_2O_2$) or bleomycin, since they induce the release of reactive oxygen species (ROS) as well as genomic DNA-damage as observed in the lungs of smokers [27]. For bleomycin and $H_2O_2$, we observed a dose-dependent induction of SA-β-galactosidase activity (Suppl. Figure 1A, B). A dose of 3.3mU/ml for bleomycin and 180uM for $H_2O_2$ was used for further experiments, since these doses induced a high percentage of senescent cells (58.8% and 61.5%, respectively) with a significant reduction in cell proliferation, both well-known characteristics of senescent cells (Suppl. Figure 1C). Next, we tested whether single (S) or repetitive (R) treatment would induce different responses in senescence-related markers. Here, we did not find any significant difference between both treatment regimes, but the repetitive treatment showed higher induction of *CDKN1A/P21* (Suppl. Figure 1D) and therefore, we continued with this treatment scheme for further experiments.

Next, we characterized the senescence phenotype of phLF derived from donor, IPF, or COPD tissue (Table 1) upon exposure to the treatment regimen for 3 and 7 days (Fig. 2A). Moreover, we included transforming growth factor beta 1 (TGF-β1), a well- known profibrotic mediator, as the third stimuli, given that previous studies showed that TGF-β1 not only promotes fibroblast activation but also senescence in vitro [27]. First, we stained for senescence-related markers: the cell cycle inhibitor *CDKN1A/P21* and the DNA damage response-related protein yH2Ax (gamma-H2A histone family member X). Here, we observed that only phLF treated with $H_2O_2$ and bleomycin displayed higher levels of these proteins compared to untreated controls (Fig. 2B). Accordingly, phLF treated with $H_2O_2$ and bleomycin showed significantly increased SA-β-galactosidase activity and decreased cell proliferation, consistent with a senescent phenotype (Fig. 2C and Suppl. Figure 2). Notably, TGF-β1 treatment increased classical fibrotic markers (Suppl. Figure 3) but did not affect the expression of *CDKN1A/P21* and yH2Ax, SA-β-galactosidase activity or cell proliferation (Fig. 2B, C and Suppl. Figure 2). The gene expression of *CDKN1A/P21* was increased again only by $H_2O_2$ and bleomycin whereas *CDKN2A/P16* was only significantly induced by $H_2O_2$ treatment (Fig. 2D). Finally, we characterized the secretion of different SASP factors. Here, we found that bleomycin significantly induced the secretion of GDF-15 while $H_2O_2$ significantly inducedMMP-3 secretion (Fig. 2E). Conversely, TGF-β1 treatment significantly induced IL-6 and PAI-1 secretion (Fig. 2E). In conclusion, $H_2O_2$ and bleomycin induced a robust senescent phenotype in all phLF characterized by cell cycle inhibition, reduced proliferation, increased SA-β-galactosidase activity, and secretion of SASP-related proteins. On the other hand, TGF-β1 treatment led to increased fibrosis-related genes and the secretion of well-known downstream mediators of the TGF-β1 signaling pathway like PAI-1 and IL-6 but did not induce a senescent phenotype in phLF.

**Senescence induction in phLF is not impacted by origin, but stimuli-specific** Next, we further analyzed the dataset we generated and asked the following two questions: 1) Do phLF from different disease background exhibits differences in susceptibility to cellular senescence? and 2) Do the senescent phenotypes induced by different stimuli differ among phLF derived from distinct disease background? To this end, we first compared the level of senescence induction in donor-, IPF, or COPD-derived phLF, respectively. Here, we did not find any significant differences in SA-β-galactosidase-induction among non-disease/disease origins (Fig. 3A, B). Similarly, we did not observe any significant difference in gene expression of three cell cycle regulators of *CDKN1A/P21*, *CDKN2A/P16*, or *TP53* (Fig. 3C).

Next, we compared the senescence signatures of phLF induced by TGF-β1, bleomycin, or $H_2O_2$ after stratification by origin of the tissue. Here, we observed that for all treatments the SA-β-galactosidase activity increased from day 3 to day 7 (Fig. 4A). Moreover, bleomycin induced the highest level of SA-β-galactosidase in phLF derived from donor and COPD tissue, while $H_2O_2$ had a higher effect in SA-β-galactosidase activity induction in IPF-derived phLF (Fig. 4A). As observed previously, TGF-β1 did not significantly induce SA-β-galactosidase activity in any of the phLF (Fig. 4A). Interestingly, both $H_2O_2$ and bleomycin induced *CDKN1A/P21* expression, whereas *CDKN2A/P16* was mainly induced after $H_2O_2$ treatment (Fig. 4B). Moreover, $H_2O_2$ and bleomycin induced the expression of *PAI-1*, *ACTA2*, and Fibronectin 1 (*FN-1*) after 7 days of culture (Fig. 4B). Conversely, TGF-β1 did not increase the evaluated senescence-related genes but predominantly induced the expression of the pro-fibrotic markers: *ACTA2*, *FN-1*, and Collagen 1 (*COL1A1*) (Fig. 4B). Finally, we characterize the secretion of selected SASP factors over the culture time. Here, we found that $H_2O_2$ and bleomycin strongly induced GDF-15 secretion on day 3, whereas IL-6 was strongly induced by TGF-β1 and bleomycin treatment (Fig. 4C). PAI-1 secretion was mainly induced by TGF-β1 and sustained over culture time (Fig. 4C). Conversely, $H_2O_2$ had the stronger effect on MMP-3 secretion after 7 days in comparison to TGF-β1 and bleomycin (Fig. 4C). In general, SASP profiles were similar between $H_2O_2$ and bleomycin as shown by an initial induction of IL-6, later induction of MMP-3 secretion, and sustained GDF-15 secretion (Fig. 4C). Finally, supporting a mainly pro-fibrotic effect, TGF-β1 only induced the secretion of PAI-1 and IL-6 (Fig. 4C). In conclusion, we observed specific senescence programs mostly depending on the senescence trigger.

## Senescent fibroblasts disrupt progenitor potential of distal alveolar epithelial cells

Senescent fibroblasts accumulate in COPD and IPF lungs. In the alveolar niche the epithelial and mesenchymal compartments closely interact but the paracrine effects of senescent cells in this environment are still understudied. To explore this, we co-cultured control or senescent phLF with distal alveolar epithelial progenitor cells in an organoid assay (Fig. 5A). Here, we also evaluated whether the modulation of stem cell function by senescent phLF depends on trigger. After 14 days, we observed formation of alveolar (small and dark and surfactant protein C (SP-C) positive), bronchiolar organoids (big with a lumen and positive for acetylated Tubulin (ACT)) and bronchoalveolar organoids (SP-C+/ACT+) in all conditions (Fig. 5C). However, phLF treated with bleomycin or $H_2O_2$ significantly reduced colony formation efficiency (CFE) of the distal lung epithelial progenitors (Fig. 5B). Moreover, bleomycin-treated phLF significantly reduced the organoid size (Fig. 5B). Finally, to characterize the cellular composition of the formed organoids, we stained them for SP-C, a marker for alveolar type 2 (AT2) cells, Keratin-8 (Krt8), a transdifferentiation marker for AT2 cells, and ACT, a marker for airway epithelium as shown in Fig. 5C. Here, we observed a similar cellular composition for $H_2O_2$ and control-derived organoids. Notably, organoids derived from the co-culture with bleomycin-treated phLF showed lower Krt8 with a higher expression of SP-C in comparison with controls and other senescence inducers, suggesting impaired AT2 activation/differentiation (Fig. 5C). In conclusion, the co-culture with senescent fibroblasts reduced stem cell capacity of alveolar epithelial progenitor cells *in vitro*.

## Discussion

Aging is the main risk factor for CLDs such as COPD and IPF and previous studies have shown that senescent cells accumulate with age [28]. Although the mechanism is not fully understood, senescent cells can evade immune clearance, thereby accumulating and promoting organ dysfunction [29–32]. Indeed, elevated levels of *CDKN1A/P21*, *CDKN2A/P16*, and SA-β-galactosidase were found in fibroblasts from both IPF and COPD lungs and therefore, have been linked to the disease pathobiology [16, 28, 33–35]. However, whether the senescent phenotype is different depending on the disease background is not well understood. Therefore, in this study we aimed to characterize the senescence of phLF from control, IPF, and COPD patients at baseline and after exposure to different senescence inducers. Finally, we used an organoid assay to study the crosstalk between epithelial and mesenchymal cells *in vitro*.

First, we characterized classical markers of senescence in phLF at baseline. Here, we found that phLF from control, IPF, and COPD had a very similar phenotype characterized by low expression of the different senescence-related markers: *CDKN1A/P21*, *CDKN2A/*P16, and *TP53* gene expression, SA-β-galactosidase activity, and secretion of SASP-related components. However, all these markers increased with prolonged culture as described for replication-induced senescence [36]. Previous studies showed that fibroblasts originated from COPD patients had an elevated senescence signature as judged by enhanced expression of *P21*/ *CDKN1A* and *CDKN2A/P16*, increased SA-β-galactosidase activity [37, 38], reduced proliferation rates [35, 38], and higher secreted levels of proteins associated with the SASP [15]. Moreover, COPD-derived fibroblasts inhibit canonical WNT-β-catenin signaling in alveolar epithelial cells by secreting WNT-5A, leading to stem cell exhaustion and impaired lung repair [39]. Similarly, phLF obtained from IPF lung tissue, showed decreased proliferation rates, increased expression of *CDKN1A/P21*, *CDKN2A/P16*, and *TP53* as well as senescence-related morphological changes [16]. However, in the present study we did not observe any major differences in senescence markers among disease origin, as reported previously [16, 35, 37, 38]. This could be explained by the different isolation protocols: in this study phLF were isolated by enzymatic digestion contrary to the outgrowth from lung tissue pieces used in other studies [37]. Furthermore, intrinsic characteristics like the smoking history or passage number, as well as different culturing conditions and media supplementation can influence senescence readouts [40]. Finally, the composition of the isolated phLF can also vary depending on different anatomical localizations such as airway [38, 41] versus whole lung [16, 35] and current isolation methods do not discriminate among the different fibroblast subtypes recently described for the lung. In the past decades, it was believed that ACTA2 + positive myofibroblasts were the main contributor for ECM deposition in the IPF lung [19]. However, recent single cell-based studies have revealed that IPF lungs have a higher heterogeneity in fibroblasts than control lungs and that these subpopulations coexist in the lung and might play distinct roles in the disease progression [17, 19]. Interestingly, the susceptibility to typical fibrotic and senescence inducers such as bleomycin, has been shown to differ among these fibroblast subpopulations in the mouse lung [19]. Therefore, the response to the stimuli used in this and other studies might be defined by the composition of the isolated and treated fibroblast population. Therefore, the development of isolation protocols for primary lung fibroblasts based on the newly described markers would help to find specific disease-relevant cellular responses of these subpopulations that could be therapeutically targeted.

Cellular senescence can be induced by several stimuli such as increased oxidative stress, caused by exposure to cigarette smoke, or genomic DNA damage, induced by chemotherapeutic agents. Therefore, we used $H_2O_2$ and bleomycin to mimic these insults in vitro. Moreover, we also included TGF-β1, since it has been shown to induce a senescent phenotype in phLF [16, 27]. In our study, only $H_2O_2$ and bleomycin induced cell cycle arrest as measured by *CDKN1A*/*CDKN2A* gene expression and other senescence-related markers such as reduced proliferation, increased SA-β-galactosidase activity, and increased secretion of GDF-15 and MMP-3 after 7 days. Notably, TGF-β1 only induced the expression of pro-fibrotic markers (*ACTA2*, *FN-1*, *COL1A1*, and *PAI-1*) as well as secretion of IL-6 and PAI-1 but did not induce a senescence phenotype defined by canonical senescence markers. This could be explained by difference in the dosage, since in previous studies much higher doses were used to observe TGF-β1-induced senescence [27]. Previous studies showed that aged individuals have around 3–4 ng/ml circulating TGF-β1 in plasma [42]. Therefore, based on results using a more physiologically relevant dose (5ng/ml), we propose TGF-β1 as a pro-fibrotic rather than a senescence stimulus.

Next, we addressed whether the susceptibility to senescence was different among the different disease origins. Here, we found that IPF-derived phLF had a trend towards a reduced response to all stimuli in comparison to Donor- and COPD-derived phLF as previously described [43]. However, as observed at baseline, we did not find any significant difference in the senescence response among the different disease origins. This could be attributed to the chosen effective treatment regimens, which consistently induce a senescent phenotype overriding the cell origin. In conclusion, the senescence response of phLF is mainly defined by the trigger, in this case DNA damage and oxidative stress, than by cellular predispositions. Interestingly, we observed similar gene expression and SASP profiles for bleomycin and $H_2O_2$ that differ from the one induced after TGF-β1. However, we also observed differences in the effect size for the tested markers. For example, the gene expression of *CDKN2A* or the secretion of MMP-3 was more pronounced on $H_2O_2$-treated phLF. Therefore, more comprehensive analysis of gene expression changes and secreted factors might be useful to better understand differences among these two senescence inducers.

Senescent cells can modulate their microenvironment in a paracrine manner by their SASP or direct cellular interactions [28, 44]. Therefore, we assessed the secretion of proteins related to inflammation and ECM deposition after induction of senescence. Here, we found that bleomycin and $H_2O_2$ induced the secretion of GDF-15, MMP-3, and PAI-1. On the other hand, TGF-β1 treatment induced the secretion only of proteins downstream of it signaling pathway: IL-6 and PAI-1. In IPF lungs, MMP-3 is secreted by different cell types, including fibroblasts, and has been linked to lung epithelium dysfunction and poor regenerative capacity as well as fibroblasts activation [45, 46]. GDF-15 and PAI-1 are well known SASP factors that also have been linked to inflammation and ECM remodeling in the diseased lung [47–49]. Interestingly, as previously described for paraquat-induced cellular senescence of lung fibroblasts [37], here we also observed that bleomycin and $H_2O_2$ decreased the expression of COL1A1 in phLF. Moreover, bleomycin and $H_2O_2$ induced the gene expression of *PAI-1* and *FN-1*. This suggests that senescent fibroblasts can contribute to ECM remodeling as seen in CLDs.

Given the fact that senescent phLF can modulate their microenvironment by secreting pro-inflammatory and ECM-related proteins, we used an organoid assay to evaluate whether co-culture with them would alter stem cell function of distal alveolar progenitor cells. Here, we found that both bleomycin and $H_2O_2$-induced senescence significantly reduced progenitor cell capacity as assessed by colony forming efficiency. However, only bleomycin significantly altered the size of the formed organoids. These could be attributed to differences in the SASP and ECM-related genes expression between bleomycin- and $H_2O_2$-induced senescence programs in phLF. Further studies focusing on a comprehensive characterization of these factors could provide insights in the specific phLF-derived factors modulating the alveolar progenitor function in vitro.

In conclusion, this study provides novel insights into the senescence phenotype of primary human lung fibroblasts exposed to disease-relevant insults. Moreover, *in vitro* organoid assays revealed that senescent phLFs modulate the regenerative capacity of the lung progenitors. Further characterization of these phenotypes using state-of-art techniques such as single cell sequencing could help elucidate the underlying mechanism that defines these senescence programs.

## Declarations

### Acknowledgments

We gratefully acknowledge the provision of human biomaterial and clinical data from the CPC-M bioArchive and its partners at the Asklepios Biobank Gauting, the LMU Hospital and the Ludwig-Maximillians-Universität München. We thank the patients and their families for their support.

### Funding

This work was funded by the German Center for Lung Research (DZL) to ML. ML acknowledges support from the Deutsche Forschungsgemeinschaft (DFG, German Research Foundation) – 512453064, Federal Institute for Risk assessment (Bundesinstitut für Risikoforschung, BfR) 60-0102-01.P588. MK acknowledges funding from NIH (NIH U554AG075931). BBL acknowledges support from the Alexander von Humbolt Foundation.

### Competing interests

The authors declare that they have no competing interests.

#### Consent for publication

Not Applicable.

#### Availability of data and materials

All data generated or analyzed during this study are included in this published article.

## Figures

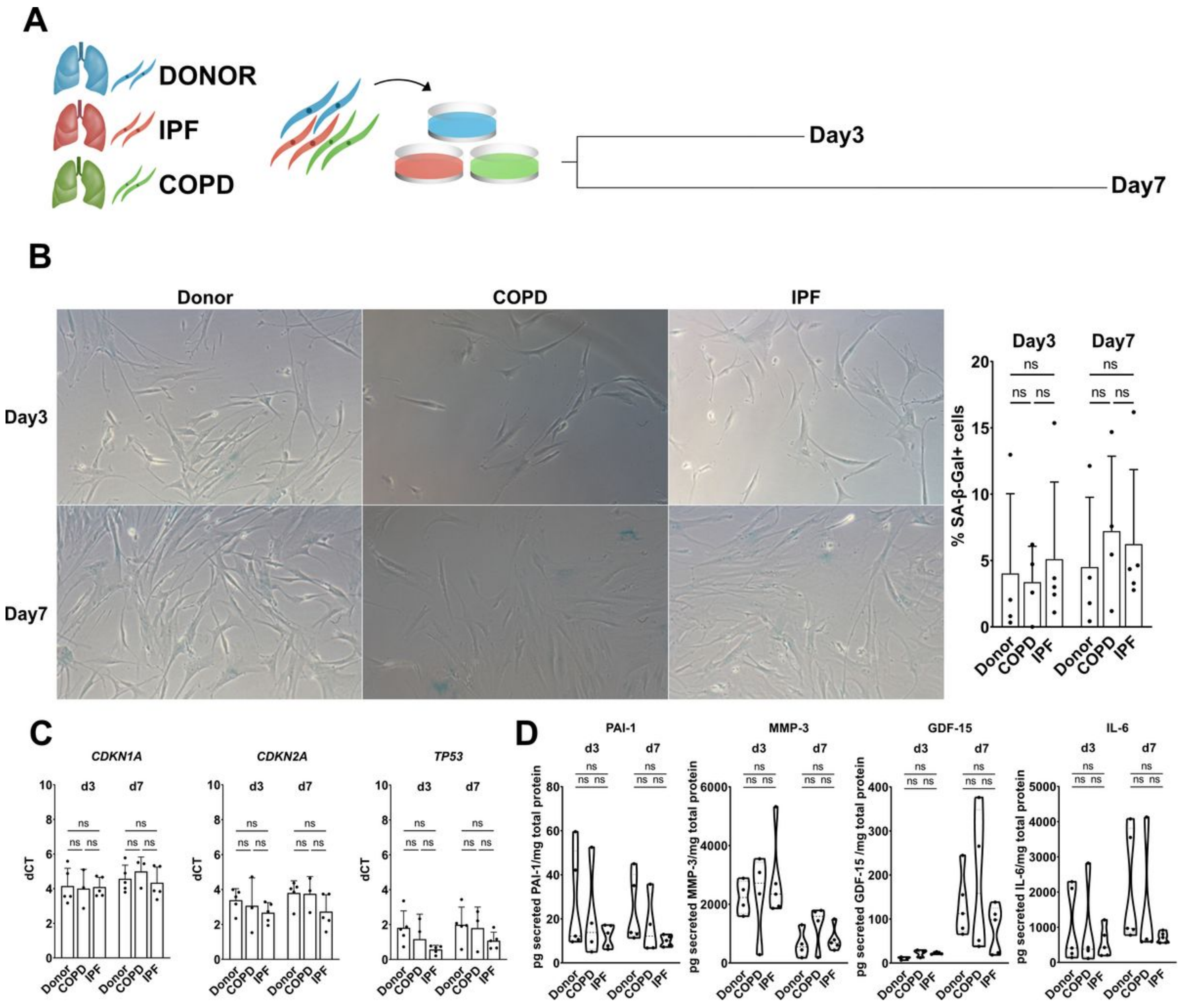

### Figure 1

**Primary human fibroblasts from different disease states do not show differences in senescence at baseline.**

A. Experimental design to characterize senescence in primary human fibroblasts from Donor, COPD, and IPF patients after short (Day3, d3) and prolonged (Day7, d7) culture. B. Representative images and quantification of SA-β-galactosidase staining of phLF from Donor, COPD, and IPF. Data points represent an average of 3 different regions of interest of at least 3 different biological replicates. D. qRT-PCR to assess gene expression of senescence-related markers (*CDKN1A, TP53, CDKN2A*) after 3 or 7 days in culture in phLF from different origins. E. ELISA of phLF supernatants that were cultured for 3 or 7 days. Data points represent different biological replicates of the concentration of each secreted protein (pg/ml)

normalized to total cell protein content (mg/ml). All p-values (<0.05) were calculated based on Kruskal-Wallis test.

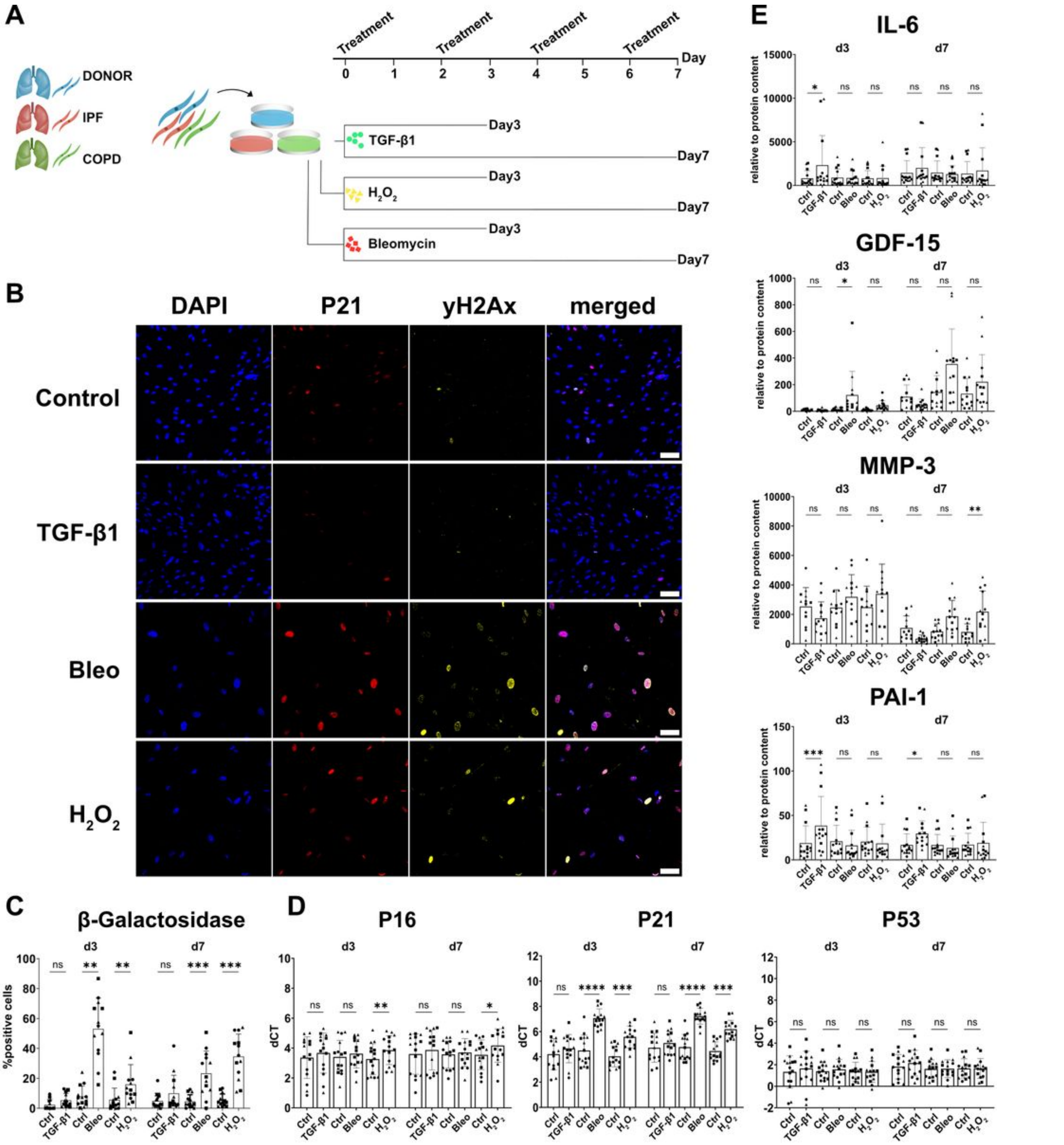

Figure 2

**Induction of senescence in primary human fibroblasts with different stimuli.**

A. Experimental design to characterize senescence induction on phLF from Donor, COPD, or IPF patients after treatment with TGF-β1, $H_2O_2$, or bleomycin, respectively. B. Representative images of immunofluorescence staining for senescence-related markers (CDKN1A/P21) and yH2Ax on phLF after 7 days of treatment with TGF-β1, $H_2O_2$, or bleomycin. C. Quantification of SA-β-galactosidase activity after 3 and 7 days of treatment. D. qRT-PCR to assess gene expression of senescence-related markers after treatment with $H_2O_2$, bleomycin, and TGF-β1 Data points represent biological replicates from donor (square), IPF (circle), and COPD (triangle). *p-value<0.05: Friedman paired-test followed by Dunn´s multiple comparisons test. E. ELISA of supernatants of phLF treated with $H_2O_2$, bleomycin, and TGF-β 1. Data points represent different biological replicates from donor (square), IPF (circle), and COPD (triangle) of the concentration of each secreted protein (pg/ml) normalized to total lysate protein content (mg/ml). *p-value<0.05: Friedman paired-test followed by Dunn´s multiple comparisons test..

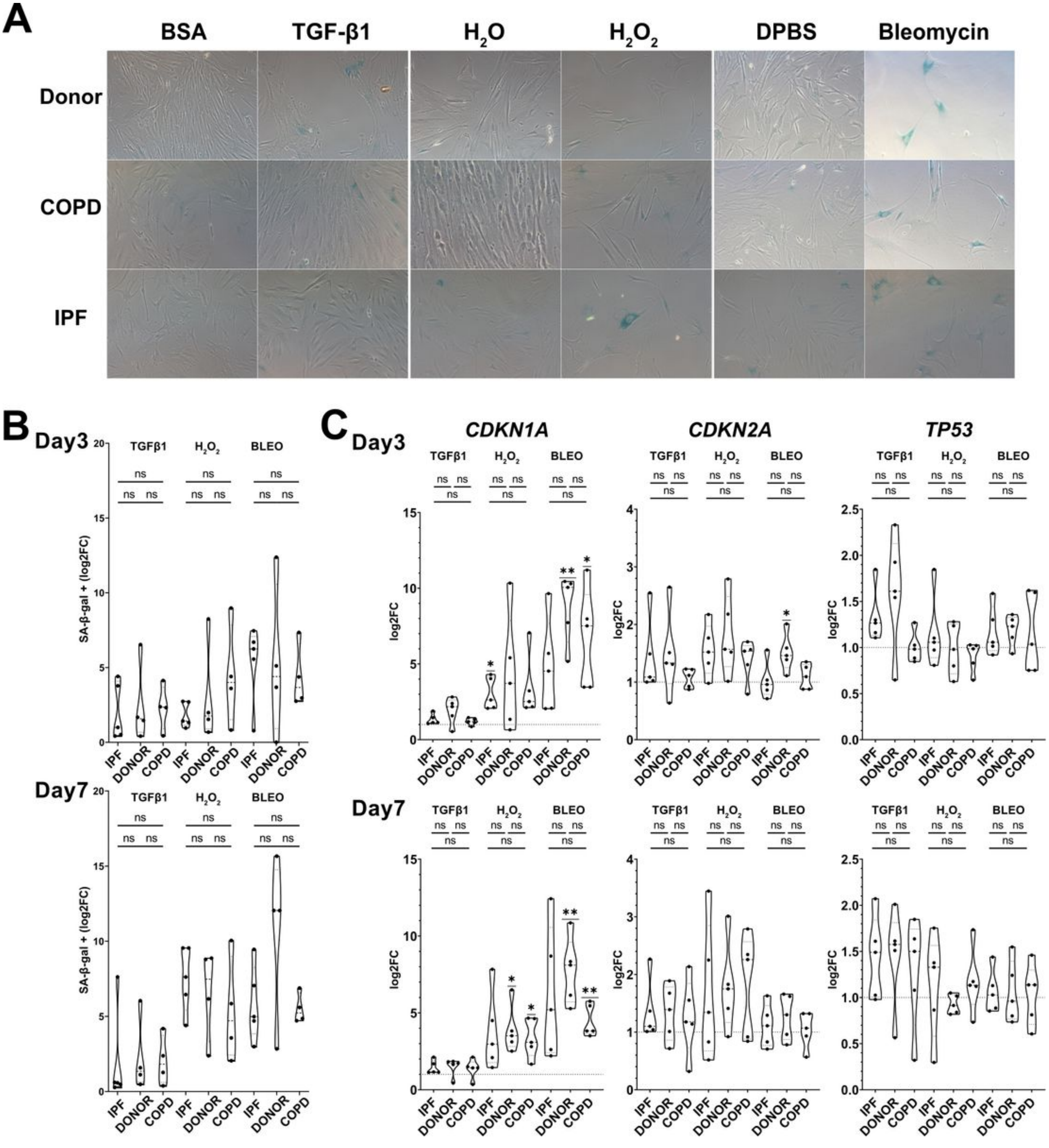

Figure 3

### No differences in senescence inducibility based on tissue origin.

A. Representative bright field images to determine senescence inducibility assay based on SA-β-galactosidase activity after 7 days of treatment with bleomycin, $H_2O_2$, or TGF-β1 in phLF from Donor, COPD, and IPF patients. B. Quantification of SA-β-galactosidase activity in phLF from Donor, COPD, and

IPF patients after 3 and 7 days of treatment with $H_2O_2$, bleomycin, or TGF-β1. C. qRT-PCR to assess gene expression of senescence-related genes (CDKN1A, CDKN2A, TP53) in phLF from Donor, COPD, and IPF patients after 3 and 7 days of treatment with $H_2O_2$, bleomycin, or TGF-β1. Origin: *p-value<0.05: Kruskal-Wallis test followed by Dunn´s multiple comparisons test. Log2FC to Ctrl: *p-value<0.05: One sample t test.

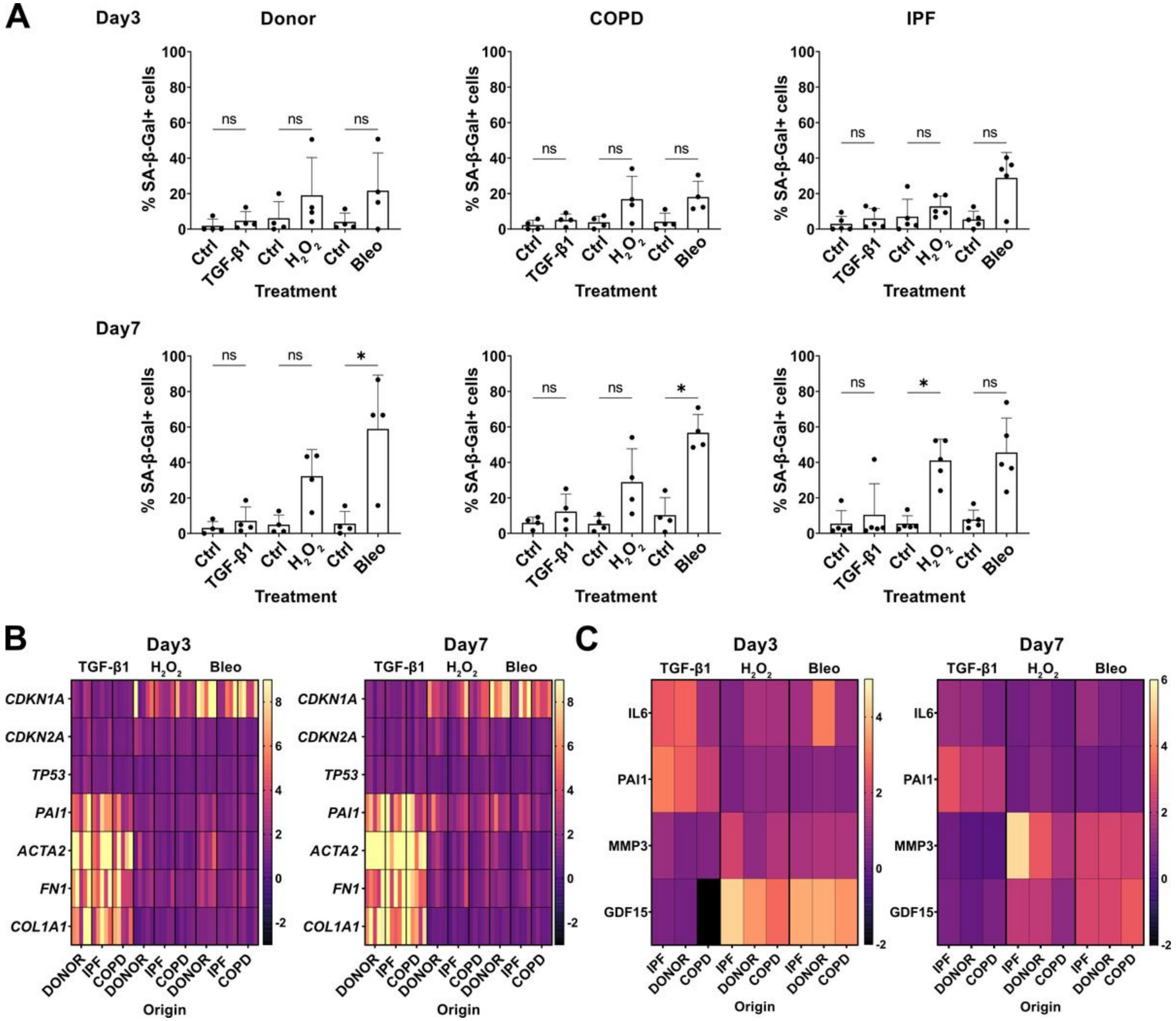

### Figure 4

#### Different stimuli induce different senescence program.

A. Quantification of SA-β-galactosidase activity induction in primary human fibroblasts from Donor, IPF, and COPD after treatment with $H_2O_2$, bleomycin and TGF-β1 for 3 and 7 days. *p-value<0.05: Kruskal-Wallis test followed by Dunn´s multiple comparisons test. B. Heatmap of qRT-PCR to assess gene expression of senescence- (*CDKN1A, CDKN2A,* and *TP53*) and fibrosis- (*ACTA2, PA-1, FN-1,* and *COL1A1*) related genes after treatment with $H_2O_2$, bleomycin, or TGF-β1 for 3 and 7 days. Single rows represent biological replicates from Donor-, IPF-, and COPD-derived phLF C. Heatmap of the SASP of Donor-, IPF-, and COPD-derived phLF as assessed by ELISA after treatment with $H_2O_2$, bleomycin, or TGF-β1 for 3 and 7 days. Single rows represent the average expression of at least 4 biological replicates. Concentration of each secreted protein (pg/ml) was normalized to total lysate protein content (mg/ml).

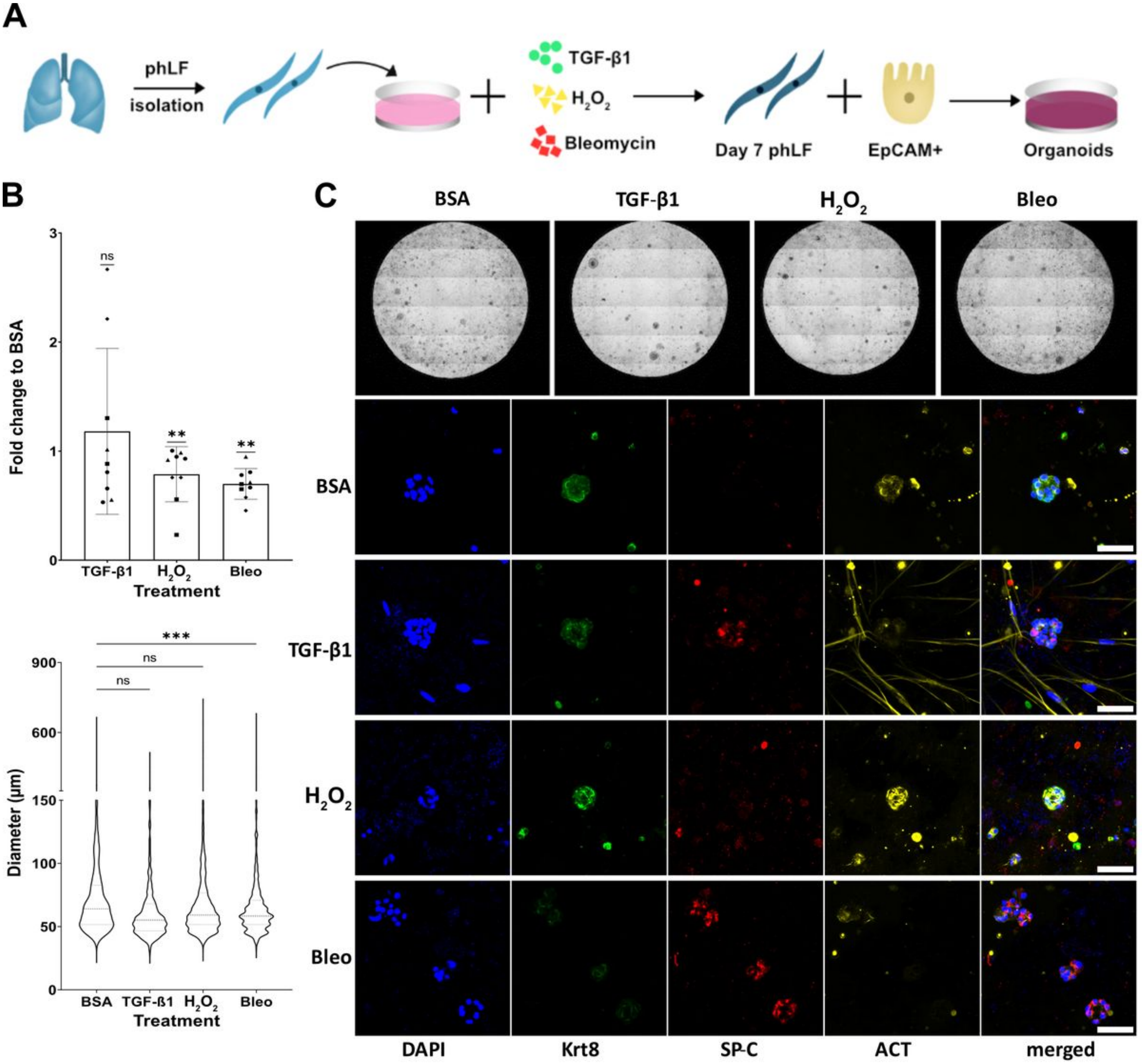

### Figure 5

**Senescent fibroblasts disrupt progenitor potential of lung epithelial cells.**

A. Experimental design. Primary mouse lung epithelial cells were isolated and co-cultured with primary human fibroblasts (Pre-treated with $H_2O_2$, bleomycin or TGF-β1 for 7 days) for 14 days in an organoid assay. B. Fold change to BSA control for colony formation efficiency (CFE, top) and average spheroid size quantification (bottom). Single points represent 4 biological replicates (marked by shape) with 2 technical replicates for each. *p-value<0.05 based on One-sample t-test (CFE) or Kruskal-Wallis test (size). C. Representative bright-field images of whole wells and fluorescence images of single organoids stained for surfactant protein C (SP-C), Acetylated Tubulin (ACT), and Keratin-8 (Krt8).

### Supplementary Files

This is a list of supplementary files associated with this preprint. Click to download.

- SupplFIGURE1.jpg
- SupplFIGURE2.jpg
- SupplFIGURE3.jpg
